# Macrophage Activation-Like Syndrome: A Distinct Entity Leading to Early Death in Sepsis

**DOI:** 10.3389/fimmu.2019.00055

**Published:** 2019-01-31

**Authors:** Eleni Karakike, Evangelos J. Giamarellos-Bourboulis

**Affiliations:** Fourth Department of Internal Medicine, Medical School, National and Kapodistrian University of Athens, Athens, Greece

**Keywords:** macrophage activation syndrome, sepsis, hemophagocytic lymphohistocytosis, ferritin, interleukins, interferon-gamma

## Abstract

Hemophagocytic lymphohistocytosis (HLH) is characterized by fulminant cytokine storm leading to multiple organ dysfunction and high mortality. HLH is classified into familial (fHLH) and into secondary (sHLH). fHLH is rare and it is due to mutations of genes encoding for perforin or excretory granules of natural killer (NK) cells of CD8-lymphocytes. sHLH is also known as macrophage activation syndrome (MAS). Macrophage activation syndrome (MAS) in adults is poorly studied. Main features are fever, hepatosplenomegaly, hepatobiliary dysfunction (HBD), coagulopathy, cytopenia of two to three cell lineages, increased triglycerides and hemophagocytosis in the bone marrow. sHLH/MAS complicates hematologic malignancies, autoimmune disorders and infections mainly of viral origin. Pathogenesis is poorly understood and it is associated with increased activation of macrophages and NK cells. An autocrine loop of interleukin (IL)-1β over-secretion leads to cytokine storm of IL-6, IL-18, ferritin, and interferon-gamma; soluble CD163 is highly increased from macrophages. The true incidence of sHLH/MAS among patients with sepsis has only been studied in the cohort of the Hellenic Sepsis Study Group. Patients meeting the Sepsis-3 criteria and who had positive HSscore or co-presence of HBD and disseminated intravascular coagulation (DIC) were classified as patients with macrophage activation-like syndrome (MALS). The frequency of MALS ranged between 3 and 4% and it was an independent entity associated with early mortality after 10 days. Ferritin was proposed as a diagnostic and surrogate biomarker. Concentrations >4,420 ng/ml were associated with diagnosis of MALS with 97.1% specificity and 98% negative predictive value. Increased ferritin was also associated with increased IL-6, IL-18, IFNγ, and sCD163 and by decreased IL-10/TNFα ratio. A drop of ferritin by 15% the first 48 h was a surrogate finding of favorable outcome. There are 10 on-going trials in adults with sHLH; two for the development of biomarkers and eight for management. Only one of them is focusing in sepsis. The acronym of the trial is PROVIDE (ClinicalTrials.gov NCT03332225) and it is a double-blind randomized clinical trial aiming to deliver to patients with septic shock treatment targeting their precise immune state. Patients diagnosed with MALS are receiving randomized treatment with placebo or the IL-1β blocker anakinra.

## Introduction

The need for adjunctive therapies in sepsis is coming from the failure of antimicrobial treatment and source control to restrain the high lethality. This led to the concept that modulation of the overwhelming host response of an infection is the strategy that needs to be followed in order to improve outcomes. It is already more than 20 years since treatment strategies with drugs aiming to modulate the exaggerated host response to an infection have started to be studied in randomized clinical trials (RCTs). A large list of agents was studied the vast majority of which was targeting pro-inflammatory mediators like cytokines. The vast majority of these RCTs failed to meet the study primary endpoint that was, for most of these RCTs, mortality after 28 days. There are two suggested explanations for the failure of these RCTs: (a) the primary endpoint is not correct since sepsis is an infectious complication so the endpoint should address the criteria for infection resolution; (b) the timing of the strategy is not correct or partly correct e.g., inhibiting an exaggerated host response may be ineffective if given to a wrong time point when the host response has ceased to exist. This last hypothesis may mostly apply for patients who develop sepsis in the field of community-acquired infections where the time from start of infection until the development of sepsis-associated organ dysfunction varies greatly from one patient to the other. The failure of RCTs with inhibitors of pro-inflammatory mediators gave birth to the concept of sepsis-induced immunosuppression that has gained much of enthusiasm the last years. We elaborate on the hypothesis that patients with sepsis can be classified into three groups regarding the mechanism of organ dysfunction:

- Patients with dominant pro-inflammatory mechanism of organ dysfunction.
- Patients with dominant anti-inflammatory mechanism of organ dysfunction.
- Patients with co-existing pro-inflammatory and anti-inflammatory mechanisms of organ dysfunction that do not remain stable and fluctuate toward the one or the other direction over time.

If this hypothesis holds true, it can be postulated that treatment of patients with a dominant pro-inflammatory mechanism of organ dysfunction mandates inhibition of pro-inflammatory mediators. In this review, we try present for the first time all published evidence demonstrating that in adults with sepsis there is a small proportion of patients who develop and maintain sepsis responses through dominant pro-inflammatory mechanisms of organ dysfunction. We also aim to review and suggest specific biomarkers of the detection of patients who develop sepsis-associated organ dysfunction with dominant pro-inflammatory mechanism and to suggest therapies that act on this pro-inflammatory mechanism. Evidence coming from studies in children is used only when evidence coming from adults is missing.

## Hemophagocytic Lymphohistocytosis: Classification and Current Concept

The situation of predominant pro-inflammatory sepsis in the adult is an expansion of our knowledge on the syndrome of hemophagocytic lymphohistiocytosis (HLH) described in children. There are two forms of HLH; the primary or familial HLH (fHLH) and the secondary HLH (sHLH). The sHLH is also known as macrophage activation syndrome (MAS). Familial HLH (fHLH) is caused by mutations affecting the cytolytic pathway of natural killer (NK) cells and CD8+ T lymphocytes. Normally, these cells recognize and kill infected cells. The majority of patients with fHLH carry mutations in *PRF1*, which encodes perforin, or in genes encoding proteins required for the docking and fusion of granules for excretory protein like *UNC13D* (encoding MUNC13–4), *STX11* (encoding syntaxin 11), and *STXBP2* (encoding syntaxin-binding protein 2). These mutations transform NK cells to become over-active and stimulate a fulminant cytokine storm leading to organ dysfunctions (1). Children are classified into HLH if they meet at least five of the eight criteria of the International Histiocyte Society (2004-HLH criteria) published in 2007: (a) fever, (b) splenomegaly, (c) cytopenia of at least two lineages; (d) fasting triglycerides ≥265 mg/dl and fibrinogen ≤150 mg/dl; (e) hemophagocytosis in the bone marrow; (f) low or absent NK-cell activity; (g) ferritin ≥500 ng/ml; and soluble CD25 ≥2,400 units/ml (2). These patients are further classified into fHLH or sHLH if they have or if they do not have positive molecular assay for one of the mutations listed above. There is large overlap between clinical signs of sHLH and of sepsis-associated organ dysfunction in children. Despite this overlap, the treatment strategy and associated prognosis are far different in children with sHLH than in children with sepsis. Management of sHLH mandates repeated cycles of chemotherapy whereas management of sepsis relies on the proper use of antimicrobials (3).

### Macrophage Activation Syndrome in the Adults: Features, Classification Criteria, and Etiology

The classification criteria for sHLH or MAS were developed by the analysis of medical records of 312 patients by three experts. The experts classified patients as positive or negative for sHLH or undetermined through a consensus approach. The main clinical characteristics associated with sHLH entered multivariate logistic regression analysis and variables independently associated with sHLH were used to construct the HSscore. This score now contains nine variables. The score may range from 0 to 317 and values >169 provide the best cut-off for classification as they have sensitivity 93% and specificity 86% allowing correct classification of 90% of cases (4). The majority of analyzed cases developed sHLH as a complication of hematologic malignancy (57% of cases), infection (25% of cases), or both malignancies and infection (4% of cases).

A total of 115 cases of patients hospitalized in Intensive Care Units (ICU) and undergoing bone marrow aspiration were retrospectively analyzed and classified using the HSscore; 71 cases were classified into confirmed sHLH. Malignancies and infection were the most common predisposing conditions complicated by HLH. The most common malignancy associated with sHLH was non-Hodgkin's lymphoma (21%) and the most common infections were those coming from Ebstein-Barr virus and from cytomegalovirus (18%) (5). These patients were admitted in the ICU with organ dysfunction mainly acute respiratory distress syndrome (ARDS, 35% of cases), circulatory shock (28% of cases) or multiple organ dysfunctions (MODS, 10% of cases). In another series of 68 analyzed cases, the most common predisposing conditions were hematologic malignancies (49% in total; of myeloid origin 13%; of B-lymphoid origin 19%; and of T-lymphoid origin 13%), and infections (33% total; viral 24% of cases) (6). The main clinical and laboratory features of the reported series of patients with sHLH published the last 5 years are provided in Table 1.

**Table 1:** Synopsis of observational case-series of adult patients with macrophage activation published the last 5 years.

**References**	**No of patients**	**Main clinical and laboratory signs (n/total patients or %)**	**Mortality (n/total patients or %)**
Barba et al. (5)	71 ICU admissions	Fever (92), hepatomegaly (44), splenomegaly (39), thrombocytopenia (45), leukopenia (10), ↑AST (71), hemophagocytosis in bone marrow (83)	38.1
Goemezano et al. (7)	17 with SLE and acute pancreatitis	Fever (94), hepatomegaly (47), splenomegaly (23), thrombocytopenia (65), leukopenia (82), ↑ triglycerides (87), ↓ fibrinogen (9), ↑AST (71)	23
Japtag et al. (8)	6	Fever (6/6), splenomegaly (6/6), pancytopenia (6/6), ↑ triglycerides (6/6), ↑ bilirubin (6/6), ↓ fibrinogen (6/6), ↑ALT/AST (6/6), hemophagocytosis in bone marrow (6/6)	2/6
Liu at al. (9)	32 with SLE	Fever (96.9), splenomegaly (56.3), anemia (87.5), thrombocytopenia (84.4), neutropenia (58.1), ↑ triglycerides (73), ↓ fibrinogen (38.7), ↑ALT (81.3), hemophagocytosis in bone marrow (100)	12.5
Schram et al. (6)	68	Fever (96), splenomegaly (73), anemia (94), thrombocytopenia (96), neutropenia (72), ↑ triglycerides (58.1), ↑ bilirubin (85), ↓ fibrinogen (62), ↑ALT (97), hemophagocytosis in bone marrow (89)	21
Schulert et al. (10)	16 with H1N1 influenza	Splenomegaly (6/16), hepatomegaly (14/16), thrombocytopenia (12/16), neutropenia (2/16), ↑ triglycerides (9/16), ↑AST (14/16), hemophagocytosis in bone marrow (13/16)	16/16

*ALT, alanine aminotransferase; AST, aspartate aminotransferase; ICU, intensive care unit; SLE, systemic lupus erythematosus; ↓, decreased; ↑, increased*.

### Pathogenesis

The mechanism of pathogenesis of sHLH/ MAS remains unclear. It seems that a trigger of persistent inflammation or antigen presentation caused by ineffective apoptosis of infected or activated or malignant cells leads, usually through continuous stimulation of Toll-like receptors (TLRs), to the activation and uncontrolled expansion of T lymphocytes, macrophages, and NK cells; this leads to marked hypercytokinemia. The reason for ineffective cytolytic killing of NK cells may rely on genetic defects in perforin-mediated cytotoxicity. Whole exome sequencing analysis has revealed a high frequency of SNP in genes causatively related to fHLH among patients with MAS developing in the field of systemic onset juvenile idiopathic arthritis (sJIA, 35.7% of cases) compared to sJIA controls (13.7% of cases; *p* = 0.098). A series of other variants involved in the trafficking and fusion of cytolytic granules of NK cells were also identified in this population. These findings can be an indication of a genetic overlap as well as a common pathogenesis pathway between sHLH/MAS and fHLH (11).

In a report of 16 patients with fatal infection by the H1N1 influenza virus, signs of hemophagocytosis were found in 13 patients. DNA sequencing for genes reported in patients with familial HLH was done. Carriage of rare variants associated with HLH was found in five patients. All five patients were carriers of mutations of the *LYST* gene; two of these five patients were also carrying mutation of the *PRF1* gene. Although the functional significance of these variants is unknown, it should be underscored that *PRF1* encodes for perforin that decreases NK cell cytotoxicity (10).

The main cytokines that play a major role in the pathogenesis of sHLH/MAS are interleukin (IL)-1β, IL-6, IL-18 and interferon-gamma (IFNγ) and one iron-binding protein, namely ferritin. Even though studies have not shown IL-1β levels to be consistently increased during sHLH (12), it could bear in mind that IL-1β is secreted locally and hence not measured. The implication of the role of IL-1β is coming indirectly both from the increase of circulating levels of the IL-1 receptor antagonist (IL-1ra) and by the therapeutic role of the IL-1β blocker anakinra in children with sHLH (13, 14).

In patients with MAS, free IL-18 concentrations significantly correlated with the clinical status and the biologic markers of MAS such as anemia (*p* < 0.001), hyper-triglyceridemia, and hyper-ferritinemia and also with markers of Th1 lymphocyte and macrophage activation, such as the elevated concentrations of IFNγ and of soluble IL-2 receptor and of the concentrations of the TNFα receptor (15).

Current knowledge on the pathogenesis of sHLH is coming from animal models in mice. These models suggest that there is no unique mechanism leading to sHLH. sHLH is commonly induced in mice after viral challenge using strains like the lymphocytic choriomeningitis virus (LCMV) and the murine cytomegalovirus (CMV). The induction of sHLH in mice by LCMV mandates the challenge into *pfp–/–* mice with homogeneous deficiency of the perforin gene. These mice develop within 10 days full clinical signs of sHLH i.e., fever, cytopenia, hypofibrinogenemia, hypertriglyceridemia and splenomegaly. Excess levels of IFNγ mediated through the activation of CD8-lymphocytes is observed (16). However, it seems that the excess levels of IFNγ do not represent a real causal link between IFNγ and sHLH. In a mouse model of sHLH induced by CMV, viral infection of mice knocked-out for IFNγ led to more severe disease phenotype with more rapid progression into death and greater cytokine production so as to indicate that excess IFNγ is a reciprocal mechanism to prevent massive tissue destruction in sHLH. This model is also characterized by microthrombi in tissue vasculature consistent with disseminated intravascular coagulation (17). Although the use of LCMV and CMV as the triggering pathogens highlights the contribution of cells of the adaptive immune system in the pathogenesis of sHLH, sHLH may be induced in mice through serial challenge with the TLR9 agonist CpG. Single challenge with CpG cannot induce sHLH but this requires serial injections in consecutive days. CpG can be a powerful stimulant of sHLH even in *Rag2*^−/−^ mice depleted by T- and B-lymphocytes. Two peaks of circulating IFNγ are observed; an early peak the first 24 h post LCMV infection and a second peak 7 days post LCMV infection. Both peaks are needed for the sHLH phenotype to be induced (18).

Girard-Guyonvarc'h and Weiss showed a causative link between free L-18 and MAS induction. Using two different mouse models of IL-18 over-activity [namely, mice over-expressing IL-18 and mice deficient in IL-18 binding protein (*Il18bp*–/–)], both research groups confirmed that high levels of free IL-18 unbound to IL-18BP increase the risk of developing MAS. Following an additional trigger through TLR9 activation both mouse models developed characteristic MAS manifestations, whereas wild-type mice did not (19, 20). Inhibition of IL-18 signaling using an antibody targeting the IL-18 receptor attenuated the severity of the MAS manifestations in *Il18bp*–/– mice. In patients suffering from auto-inflammatory disorders and hyper-ferritinemia a dramatic correlation of the risk for the development of MAS risk with the chronic (sometimes lifelong) elevation of free IL-18 was found (20). In a specific type of MAS associated with gain-of-function mutations in *NLRC4* that cause inflammasome hyperactivity, IL-18 was derived from the intestinal epithelium.

Increased ferritin is a common denominator of sHLH and of some cases of septic shock (21). It is not fully elucidated whether it is a bystander highly increased or a pro-inflammatory mediator *per se*. Mortality analysis was done in 405 adult patients with ultra-elevated ferritin >5,000 ng/ml. Overall mortality 30 days and 6 months after index serum ferritin measurement was 32 and 50%, respectively. For patients with serum ferritin between 5,000 and 10,000 ng/ml, mortality was 27% after 30 days and 49% after 6 months. For patients with serum ferritin between 10,000 and 20,000 ng/ml, mortality was 41% after 30 days and 50% after 6 months. For patients with serum ferritin between 20,000 and 40,000 ng/ml, mortality was 41% after 30 days and 52% after 6 months. For patients with serum ferritin >40,000 ng/ml, mortality was 52% after 30 days and 57% after 6 months. Sepsis was not identified as a cause of hyperferitinaemia among patients who were also suffering from malignancies (22).

Ferritin is an iron storage protein including heavy (H) and light (L) subunits. In a study evaluating bone marrow biopsies of patients with MAS, H-ferritin, IL-1β, TNFα, and IFNγ were significantly increased. Furthermore, an increased number of CD68+ /H-ferritin+ cells and an infiltrate of cells co-expressing H-ferritin and IL-12, suggesting an infiltrate of M1 macrophages, were found. H-ferritin levels and CD68+ /H-ferritin+ cells were correlated with hematological involvement of the disease, serum ferritin and C-reactive protein (23). Ferritin synthesis is up-regulated in response to hemoxygenase-1 activation to remove any iron that could exacerbate oxidative stress. Ferritin also acts as an antiapoptotic agent in ischemia-reperfusion injury. It may be the case that ferritin acts like a danger-associated molecular pattern (DAMP) ending with the stimulation of NF-κB and the over-production of IL-1β.

**Figure 1:**
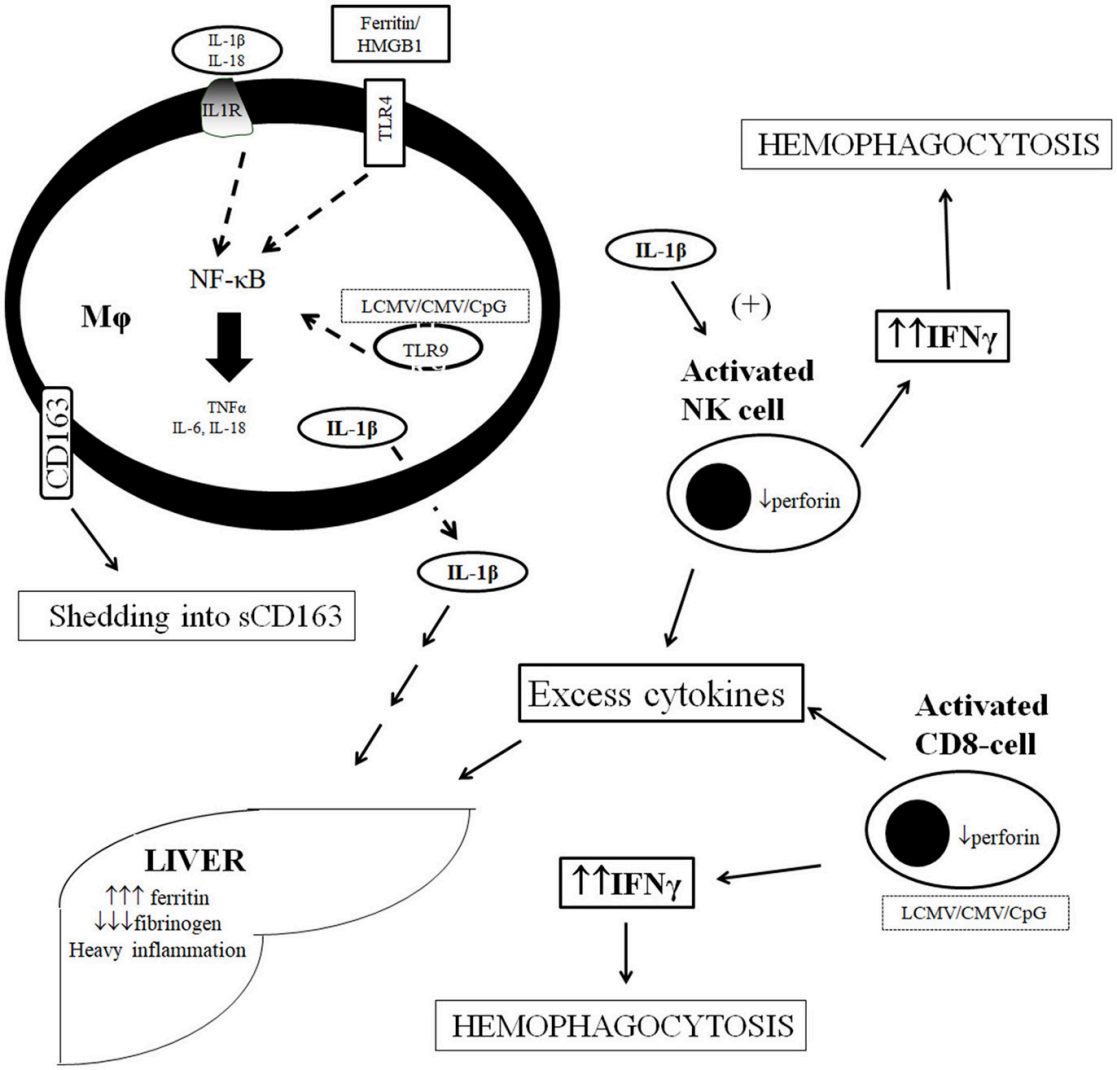
Current concept of pathogenesis of macrophage activation syndrome in sepsis. Over-production of IL-1β is effected through the stimulation of TLRs by ferritin and HMGB1 and by the *per se* autocrine effect of IL-1β on IL-1R at the macrophage level. Stimulation leads to cytokine storm and shedding of CD163 from macrophage cell membrane. Over-produced cytokines stimulate further ferritin production by the liver and liver dysfunction whereas IL-1β leads to over-production of IFNγ by NK cells leading to hemophagocytosis. Excess cytokine production may results independently by distirbances in NK cell and CD-cell function leading to their over-activation. CMV, cytomegalovirus; CpG, bacterial oligonucleotides; IL, interleukin; IL-1R, receptor of IL-1; LCMV, lymphocytic choriomeningovirus; Mϕ, macrophage, NF-κB, nuclear factor kappa B; NK, natural killer; TLR, Toll-like receptor; TNFα, tumor necrosis factor-alpha.

A schematic representation of the pathogenesis of sHLH/MAS in sepsis is shown in Figure 1.

### Biomarkers

Apart from ferritin mentioned above, there is no consensus on which the best diagnostic biomarkers for the diagnosis of sHLH/MAS are. Soluble CD163 is the soluble counterpart of the CD163 receptor for the hemoglobin-haptoglobin complex on the cell membrane of M2 macrophages. Increases of sCD163 signify intense differentiation of M2 macrophages through the alternate pathway related to phagocytic activity. In a recent study, sCD163 was measured in the serum of 63 patients with sJIA. Concentrations were greater among patients with active sJIA associated with macrophage activation and they were decreased upon disease remission. Positive associations were found with circulating IL-18 and ferritin and with liver aminotransferases (24). Similar increases of sCD163 were reported for 34 patients with adult-onset Still'disease (AOSD) in whom sCD163 was elevated at similar levels as for 16 patients with sepsis. Immunohistochemistry of lymph nodes and of the tonsils revealed equal distribution of sCD163 in both B-rich and T-rich areas contrary to H-ferritin that was mainly expressed in B-rich areas (25).

Although not conducted in adults, a recent analysis supports concentrations of fibrinogen in plasma lower than 150 mg/dl as suggestive of the presence of sHLH/MAS. More precisely, 190 admissions of patients aged <21 years were retrospectively analyzed. Patients were split into those with hypofibrinogenemia (≤150 mg/dl, *n* = 38) and into those with normal of elevated fibrinogen (>150 mg/dl, *n* = 154) based on available fibrinogen measurements at the beginning of follow-up. A composite endpoint, namely, complicated course, was selected by the authors as the primary study endpoint. This was composed from the presence of at least one unfavorable outcome i.e., death after 28 days or ≥ two organ dysfunctions the first 7 days. This primary endpoint was met in 73.7% of patients with hypofibrinogenemia compared to 29.2% of comparators (*p* < 0.0001). Patients were also classified into sHLH/MAS based on the 2004-HLH criteria (2). The proportion of sHLH/MAS was 15.8% in the group of hypofibrinogenemia whereas it was 1.3% among the comparators (*p* < 0.0001) (26).

In a recent retrospective study, positron emission tomography/computed tomography (PET/CT) with ^18^F-fluorodeoxyglucose uptake was conducted in 34 patients classified with MAS using the HSscore. Fifteen patients with sepsis scoring negative for the HScore were studied as comparators. The spleen to liver maximal uptake value (SLR_max)_ was greater in MAS than sepsis. The best discriminator was for values >1.31 associated with odds ratio 8.175 for MAS. Patients with SLR_max_ >1.72 died earlier (27). Although these findings point the role of the spleen as a major lymphoid and macrophage pool for MAS, they also underscore the diagnostic and prognostic role of FDG-PET/CT.

## Macrophage Activation Syndrome and Sepsis: the Birth of a Hypothesis

As analyzed above among infections causing MAS, bacteria are the least common causes whereas viruses are the major causes. The dominant feature of MAS is the over-activation of tissue macrophages for the release of a storm of cytokines leading to rapidly progressing organ dysfunction where pancytopenia, tissue hemophagocytosis, hepatobiliary dysfunction (HBD), disseminated intravascular coagulation (DIC), and dysfunction of the central nervous system predominate. Macrophage activation syndrome (MAS) often leads to early death. The hallmark of pathogenesis is the over-production of IL-1β by tissues macrophages. IL-1β acts through an autocrine way on macrophages leading to a vicious cycle of further cytokine production and exaggerated inflammation.

Anakinra is the recombinant humanized form of IL-1 receptor antagonist that inhibits both IL-1β and IL-1α. In one phase 3 RCT conducted 25 years ago, anakinra was administered intravenously in 906 patients with severe sepsis. The study was prematurely stopped for futility (28). Twenty years after the conduct of this trial, clinical data of enrolled patients were retrospectively reviewed. Among enrolled patients, those presenting both with HBD and DIC were considered to have traits of MAS. A total of 43 patients were classified with MAS, 26 of whom were treated with anakinra and 17 with placebo; 28-day mortality was 35 and 65%, respectively and this difference was statistically significant (*p*: 0.0006) (29). Although these results were inconclusive, they triggered the concept that in a small fraction of sepsis patients pro-inflammatory phenomena predominate and that treatment may be associated with better outcomes.

## Macrophage Activation-Like Syndrome in Sepsis: Does This Exist?

In order to investigate if a situation like MAS exists in sepsis, we run an analysis of 5,121 patients registered in the prospective cohort of the Hellenic Sepsis Study Group using a test and validation approach. We set specific criteria of classification (presented in Table 2) and we called this syndrome macrophage activation-like syndrome (MALS) because routine bone marrow biopsy was not available for the patients. According to the criteria, patients with sepsis (as defined by the Sepsis-3 definition) and with HSscore more than 151 or who were presenting with both HBD and DIC were classified into MALS. 3.7% of the test cohort (*n* = 3,417 patients) and 4.3% of the validation cohort (*n* = 1,704) were classified into MALS. Macrophage activation-like syndrome (MALS) was an independent variable associated with early death after 10 days (odds ratio 1.86 in the test cohort; 2.81 in the validation cohort) (30).

**Table 2:** Suggested classification criteria for macrophage activation-like syndrome in sepsis used in the manuscript by Kyriazopoulou et al.

**Sepsis** (defined as total SOFA score ≥2 points for new admissions or as increase of total SOFA score ≥2 points for hospitalized patients)			
**+ either positive HSscore or both HBD and DIC**			
**HSscore (more than 151 points are needed)**		**HBD**	
	**Points**	Presence of at least 2 of the following:	
• Infection by HIV or long term immunosuppressive treatment e.g., cyclosporine, glucocorticoids, azathioprine	18	• Serum bilirubin > 2.5 mg/dl	
		• Aspartate aminotransferase ≥2 × upper normal limit	
		• International normalized ratio (INR) >1.5	
• Core temperature			
<38.4°C	0		
38.4–39.4°C	1	**DIC score (more than 5 points are needed)**	
>39.5°C	2		**Points**
• Organomegaly		• Platelet count (/mm^3^)	
Hepatomegaly or splenomegaly	1	<100,000	1
Hepatomegaly and splenomegaly	2	<50,000	2
• Number of cytopenias		• D-dimers	
1 lineage	0	No increase	0
2 lineages	24	Moderate increase	2
3 lineages	34	Strong increase	3
• Ferritin (ng/ml)		• Prothrombin time	
<2,000	0	<3 s	0
2,000–6,000	35	3–6 s	1
>6,000	50	>6 s	2
• Triglycerides (mmol/l)		• Fibrinogen (g/l)	
<1.5	0	>1	0
1.5–4	44	<1	1
>4	64		
•Fibrinogen (mg/l)			
>2.5	0		
≤ 2.5	30		
• Serum aspartate aminotransferase (U/l)			
<30	0		
≥30	19		

*DIC, disseminated intravascular coagulation; HBD, hepatobiliary dysfunction; HIV, human immunodeficiency virus; HS, hemophagocytosis; SOFA, sequential organ failure assessment; <, less than; >, more than; ≤, less than or equal to; ≥, more than or equal to*.

We developed ferritin measurement of the first 24 h as a diagnostic biomarker of MALS. Ferritin has already been proposed by others to be the diagnostic hallmark of MAS. We selected a cut-off concentration of 4,420 ng/ml that was associated with 97.1% specificity and 98% negative predictive value for diagnosis. The reason of selection of this cut-off was the high diagnostic specificity that was necessary for the biomarker to be used for treatment guidance. Twenty-eight-day mortality for patients having ferritin more than 4,420 ng/ml was 66.7% in the test cohort and 66.0% in the validation cohort. This was 52.9% in a separate cohort of 109 Swedish patients with severe sepsis/septic shock. Hyperferritinemia was accompanied by elevated serum concentrations of IL-18, IFNγ and of sCD163 and by decrease of the ratio IL-10/TNFα pointing toward the pro-inflammatory nature of these patients. Ferritin concentrations started to decrease within the first 48 h among survivors; more than 15% decrease was associated with 0.13 odds ratio for favorable outcome.

The above findings are compatible with the existence of a small proportion of 3–4% of sepsis patients who have MALS at sepsis onset. However, they do not provide evidence if MALS can develop later during the course of sepsis. Stimulation of pro-inflammatory innate responses can also come from DAMPs. One of these DAMPs is the non-histone nuclear protein high mobility group box-1 (HMGB1) that is released after cell destruction. HMGB1 can stimulate tissue macrophages. Serial measurements of circulating HMGB1 after sepsis onset allows classification of patients into those who present early peak of HMGB1 over the disease course (i.e., the first 48 h from sepsis onset) and into those who present late peak of HMGB1 over the disease course (i.e., after seven days from sepsis onset). Mortality after 28 days was 9.6 and 26.9%, respectively (*p*: 0.026). This late peak of HMGB-1 was accompanied by increased serum levels of ferritin and IFNγ implying that patients with late peak of HMGB1 were entering into a mechanism resembling MALS. Surprisingly, the late peak acted synergistically with the history of chronic comorbidities with a pro-inflammatory component, namely type 2 diabetes mellitus, chronic heart failure and chronic renal disease, and increased substantially the risk for 28-day mortality (31).

Serum levels of HMGB1 were measured in the sera of children with MAS developing as a complication of sJIA or of systemic lupus erythematosus (SLE). Samplings were done at MAS onset and repeated upon clinical improvement 2 and 8 weeks after start of etoposide treatment. HMGB1 was found increased and it was decreasing over-time (32). Similar time kinetics were also found for ferritin, IL-18 and IFNγ, that have already been described to be the combination of elevated cytokines that are characteristic of pro-inflammatory sepsis (30). This observation in children complements the observation in sepsis patients with late HMGB1 peaks and elevated ferritin and IFNγ accompanied by high mortality suggesting that HMGB1 can be a stimulator of MAS. What remains to be explained is how the late peak of HMGB1 acts synergistically with chronic comorbidities to prime unfavorable outcome. One explanation may come from the results of the CANTOS trial. In this RCT, survivors from a first myocardial infarct were randomized to blind treatment with placebo or with escalating doses of the IL-1β blocker canakinumab. Results showed substantial decrease of the risk of secondary cardiovascular death (33). This clinical benefit was achieved through IL-1β inhibition pointing toward tissue macrophage-derived elevated IL-1β production as a common denominator for situations with atherosclerosis like type 2 diabetes mellitus, chronic heart failure, and chronic renal disease. This sterile chronic pro-inflammation is definitively mediated by DAMPs. Since DAMPs like HMGB1 also participate in septic phenomena, it should not be surprising that a state of chronic stimulation of tissue macrophages synergizes with a sepsis state of peak in HMGB1.

The significance of elevated ferritin as a prognostic biomarker in sepsis, albeit not in adults, was recently published by Carcillo et al. (34). In a series of 100 consecutive admitted children with severe sepsis, a contingency Table was built using ferritin and CRP cutoffs of 1,980 ng/ml and 4.08 mg/dl, respectively. Patients with both markers above the cut-offs were classified with high mortality risk of 46.15%; those with one of the two biomarkers above the cut-offs as intermediate risk with mortality ranging between 0 and 4.65%; and those with both biomarkers below the cut-offs as low-risk with 0% mortality. High-risk defined by the two biomarkers was the only variable independently associated with 28-day mortality in the pediatric ICU (odds ratio = 9.58; *p* = 0.019; Z statistic = 2.35); whereas, bacterial infection (*p* = 0.07; Z statistic = 1.83), age (*p* = 0.96; Z statistic = 0.05), cancer diagnosis (*p* = 0.45; Z statistic = 0.76), PRISM score (*p* = 0.88 Z; statistic = 0.88), and maximum OFI (*p* = 0.13; *Z* statistic = 1.52) did not (34).

## Current Treatment Modalities and the Need for Large-Scale Randomized Clinical Trials

There is no gold-standard of treatment of sHLH/MAS. As a rule, treatment modalities like etoposide, glucocorticosteroids, anakinra, and intravenous immunoglobulins that are already in use in children (35) are also in use in adults. Available evidence is coming from case-series with limited number of patients. In a study of 25 critically ill patients with respiratory failure and/or hemodynamic instability following influenza A/H1N1 infection in Germany, nine patients were found to develop sHLH and consequently MODS (36). Of them, six were assigned to treatment; four patients with etoposide/ dexamethasone and two patients with steroids; only one patient survived. Although it is difficult to distinguish treatment failures from treatment delay or treatment harm, eight deaths occurred as a result of uncontrolled disease progress leading to MODS. After a retrospective survey in 19 adults with sHLH, Kumar et al. proposed that personalized decision making is warranted depending on the clinical presentation and course of disease. In their analysis, the majority of patients received anakinra, cyclosporine, intravenous immunoglobulins, and steroids. After excluding patients with hematologic malignancies, survival was 88% (37).

Although no data exist for adults, an open-label prospective study has been conducted in children with sHLH randomized to high-volume hemofiltration (HVHF) (*n* = 17) or not (*n* = 16) with a filter that was anticipated to remove pro-inflammatory mediators. Although the study was not powered for mortality, this was 29.4 and 56.3%, respectively, after 28 days; albeit this difference was not statistically significant. However, among patients receiving HVHF significant decrease over-time of ferritin, TNFα, and IL-6 was found; increase of NK cell activity was found as well (38).

The realm for the management of sHLH/MAS in adults mandates the results of one large-scale randomized clinical trial (RCT). Table 3 summarizes the features of on-going clinical trials for the development of diagnostic biomarkers and for the management of sHLH/MAS in adults. The real flare coming from this Table is that our understanding of sHLH/MAS is still in its infancy since we are still in need of tools to recognize this syndrome whereas the majority of on-going clinical trials are open-label and single-arm. Only one of these trials is featuring in a population of patients with sepsis. PROVIDE (personalized randomized trial of validation and restoration of immune dysfunction in severe infections and sepsis; EudraCT 2017-002171-26; ClinicalTrials.gov identifier NCT03332225) is a RCT of personalized approach in sepsis. This is a double-blind, double-dummy phase 2 study in which enrolled patients are suffering from septic shock due to lung infection, primary bacteremia and acute cholangitis and laboratory signs of MALS or hypo-inflammation on two serial time measurements. Ferritin above 4,420 ng/ml is the diagnostic tool of MALS and CD14/HLA-DR <30% in the absence of high ferritin is the diagnostic tool of hypo-inflammation. The study is on-going in 14 study sites in Greece and the primary endpoint is 28-day mortality. Patients with MALS are randomized into treatment with placebo or anakinra.

**Table 3:** Summary of on-going clinical trials for the diagnosis and management of secondary hemophagocytic lymphohistiocytosis (HLH) and macrophage activation syndrome (MAS) in adults.

**Registration at ClinicalTrials.gov**	**Design**	**Title**	**Intervention**	**Study population**	**Anticipated enrolment**	**Primary endpoint**
NCT03510650	Prospective diagnostic	Diagnostic biomarkers for adult HLH in critically ill patients	Blood sampling for cytokine panel, EBV, and CMV viral loads, microRNAs	Adults with suspected or diagnosed HLH or sepsis	50 patients with sepsis and 50 patients with sHLH	Incidence of HLH in intensive care units based on HLH-2004 criteria
NCT03259230	Prospective, case-control	Diagnostic biomarkers for sHLH	Blood sampling	Adults and children with HLH at the context of malignancy	25 patients and 25 controls	Inflammatory markers and genetic variants in malignancy-associated HLH
NCT02631109	Single-arm, open-label	L-DEP regimen as a salvage therapy for refractory EBV-induced HLH	L-DEP + pegaspargase + (doxorubicin or etoposide + methylprednisolone)	>14 years with EBV-induced sHLH	120 patients	% treatment response
NCT02780583	RCT	Treatment of MAS with anakinra	• Placebo + methylprednisolone • Anakinra + methylprednisolone	• Children or adults with sJIA and two or more of: ↓platelets; ↑AST; ↓WBCs; ↓fibrinogen; or three or more of combined clinical / laboratory criteria (↓ platelets; ↑AST, ↓WBCs; ↓fibrinogen; central nervous system dysfunction; hemorrhages; hepatomegaly) • Children or adults without sJIA and ferritin >2,000 ng/ml and 3 out of the following: bicytopenia; ↑fasting triglycerides; splenomegaly; ↑ALT or AST; fever; ↓fibrinogen < 1.5 g/L or INR >1.5 or D-dimers >500 ng/ml	40 patients	Number of acquired infections and deaths after 72 h
NCT03332225	Double-dummy RCT	A trial of validation and restoration of immune dysfunction in severe infections and sepsis	• Placebo • Anakinra or rhIFNγ	• Adults with septic shock due to lung infection or primary bacteremia or acute cholangitis • Ferritin >4,420 ng/ml (MALS) or low ferritin and CD14/HLA-DR < 30%	278 patients	Mortality after 28 days
NCT02400463	Single-arm, open-label	Pilot study of ruxolitinib in secondary HLH	Ruxolitinib	Adults with at least 5 of the following): fever, splenomegaly, cytopenia, hypertriglyceridemia, or hypofibrinogenemia, tissue demonstration of hemophagocytosis, ↓ NK cell activity, ferritin ≥3,000 ng/ml, soluble IL-2 receptor >2,400 U/ml	10 patients	Survival after 2 months
NCT03533790	Single-arm, open-label	DEP-ruxolitinib regimen as a salvage therapy for refractory/relapsed HLH	DEP-Ru	Age 1–70 years old who meet HLH-2004 diagnostic criteria	80 patients	% treatment response
NCT02385110	Single-arm, open-label	Alemtuzumab or tocilizumab in combination with etoposide and dexamethasone for the treatment of adult patients with HLH	• Alemtuzumab + etoposide + dexamethasone • Etoposide + dexamethasone + tocilizumab	Adults with HLH-2004 criteria	40 patients	Response rate

*ALT, alanine aminotransferase; AST, aspartate aminotransferase; CMV, cytomegalovirus; EBV, Ebstein-Barr virus; IL, interleukin; INR, international normalized ratio; NK, natural killer; RCT, randomized clinical trials; sJIA, systemic juvenile idiopathic arthritis; WBCs, white blood cells*.

## Conclusions

The above analysis shows that we are still in a very early stage of our understanding of the frequency of sHLH/MAS in critically ill patients with sepsis. Current evidence suggests that about 3–4% of patients are in that state dominated by pro-inflammatory host responses. Our suggestion of using ferritin cut-offs >4,420 ng/ml does not guarantee the miss of great many of these patients but is just focusing on the use of a biomarker cut-off associated with great specificity for diagnosis. Future mandates the development of better diagnostic tools. Their development will favor the conduct of RCTs to tailor individualized needs.

## Author Contributions

All authors listed have made a substantial, direct and intellectual contribution to the work, and approved it for publication.

### Conflict of Interest Statement

EG-B has received honoraria (paid to the University of Athens) from AbbVie USA, Abbott CH, Biotest Germany, Brahms GmbH, InflaRx GmbH, the Medicines Company; MSD Greece and XBiotech Inc. He has received HemoSpec by FrameWork Program 7 and by the ITN-Marie Curie grant European Sepsis Academy. EK is funded by the ITN-Marie Curie grant European Sepsis Academy. He has received independent educational grants from AbbVie, Abbott, Astellas Pharma, AxisShield, bioMérieux Inc, InflaRx GmbH, the Medicines Company, and XBiotech Inc. The remaining author declares that the research was conducted in the absence of any commercial or financial relationships that could be construed as a potential conflict of interest.
